# Impact of Flap Thickness on Refractive Outcomes and Corneal Biomechanics Following Myopic Femtosecond Laser-Assisted LASIK

**DOI:** 10.3390/jcm15051923

**Published:** 2026-03-03

**Authors:** Joanna Wierzbowska, Marcin Smorawski, Janusz Sierdziński, Łukasz Stróżecki, Anna Maria Roszkowska

**Affiliations:** 1 Department of Ophthalmology, Military Institute of Medicine—National Research Institute, 128 Szaserów Str., 04-141 Warsaw, Poland; 2 Optegra Eye Clinic, 02-366 Warsaw, Poland; 3 Doctoral Study Program, Military Institute of Medicine, 04-141 Warsaw, Poland; 4 Department of Bioinformatics, Medical University of Warsaw, 00-581 Warsaw, Poland; janusz.sierdzinski@wum.edu.pl; 5 Department of Anesthesiology and Intensive Care, Medical University of Wroclaw, 50-556 Wroclaw, Poland; 6 Ophthalmology Clinic, Department of Biomedical Sciences, University of Messina, 98124 Messina, Italy; aroszkowska@unime.it; 7 Ophthalmology Section, Faculty of Medicine and Health Sciences, Andrzej Frycz Modrzewski Kraków University, 30-705 Kraków, Poland

**Keywords:** laser in situ keratomileusis, corneal biomechanics, flap thickness, corneal hysteresis, corneal resistance factor, myopia, Ocular Response Analyzer

## Abstract

**Background/Objectives**: Femtosecond laser-assisted LASIK (FS-LASIK) is currently the most commonly performed procedure for the correction of myopia and myopic astigmatism. However, it inherently weakens the biomechanical integrity of the cornea due to flap creation and stromal ablation. This prospective study aimed to compare refractive and corneal biomechanical parameters after myopic FS-LASIK with different flap thicknesses and to identify parameters that may influence the change in corneal biomechanics after surgery. **Methods**: A total of 246 eyes were enrolled and divided into two groups based on flap thickness: 110 µm (n = 129) and 140 µm (n = 117). All procedures were performed using a femtosecond LDV Ziemer laser and standardized ablation profiles with similar ablation depths. Visual acuity, refractive outcomes, and corneal biomechanical parameters—corneal hysteresis (CH) and corneal resistance factor (CRF)—were assessed preoperatively and during a 6-month follow-up using the Ocular Response Analyzer (ORA). Multivariate regression analysis was used to identify predictors of biomechanical change. **Results**: The groups did not differ in preoperative values of the mean refractive spherical equivalent, keratometry, central corneal thickness, CH and CRF. At 6 months, both groups achieved comparable refractive outcomes, with no significant differences in uncorrected or corrected distance visual acuity, efficacy index and safety index. However, the thicker flap group exhibited significantly greater reductions in CH (−2.89 vs. −2.04 mmHg, *p* < 0.05) and CRF (−3.61 vs. −2.77 mmHg, *p* < 0.05), as well as greater biomechanical weakening per micron of ablation. Multivariate regression identified anterior weighted biomechanical index (AWBI) and flap thickness as the strongest predictors of CH reduction, while flap thickness, residual stromal bed thickness, ablation depth, and central corneal thickness contributed to CRF changes. **Conclusions**: While FS-LASIK with both flap thicknesses achieved equally effective visual outcomes, thicker flaps were associated with significantly greater biomechanical weakening. Flap thickness had a stronger influence on corneal biomechanics than ablation depth. These findings support consideration of flap thickness in surgical planning to optimize corneal biomechanical stability.

## 1. Introduction

The introduction of the microkeratome-assisted laser in situ keratomileusis (MK-LASIK) method in the 1990s initiated the development of laser stromal techniques, including femtosecond laser-assisted LASIK (FS-LASIK) and keratorefractive lenticule extraction (KLEX), and led to a substantial increase in the number of vision correction procedures performed [1]. FS-LASIK is currently considered the gold standard in corneal refractive surgery and remains the most commonly performed refractive procedure worldwide [2].

A comprehensive understanding of corneal biomechanics is essential for identifying subtle corneal abnormalities and supporting individualized treatment planning, ultimately contributing to improved postoperative visual outcomes [3,4,5]. Growing evidence indicates that laser in situ keratomileusis (LASIK) alters corneal biomechanical properties as measured by the Ocular Response Analyser (ORA) and Corvis ST [6,7,8,9]. An ex vivo experimental study demonstrated that vertical side-cut incisions through the corneal stroma, rather than horizontal lamellar dissections, play a major role in compromising the structural integrity of the cornea during LASIK procedures [10].

Clinical studies and meta-analyses consistently report a greater postoperative reduction in corneal hysteresis (CH) and corneal resistance factor (CRF) following MK-LASIK or FS-LASIK procedures, compared to SMILE and PRK, particularly in patients treated for myopia and myopic astigmatism [8,9]. Importantly, the vast majority (96%) of documented postoperative ectasia cases have occurred in eyes that underwent procedures involving corneal flap creation [11,12]. However, a recent meta-analysis by Chen et al. [13], which included both ORA and Corvis ST measurements, found no significant difference in corneal biomechanical parameters between SMILE and FS-LASIK at three months postoperatively.

Moreover, myopic LASIK has been associated with a greater reduction in ORA-derived corneal biomechanical parameters than hyperopic LASIK, despite comparable flap thickness and attempted ablation volumes. The higher naive ocular rigidity reported in hyperopic eyes, along with the ablation profile concentrated in the thicker paracentral and peripheral regions of the corneal stroma, may account for the significantly lower incidence of postoperative ectasia in hyperopic LASIK patients [14].

Several factors have been identified as influencing postoperative corneal biomechanical alterations. These include the preoperative intrinsic properties of the cornea, the percentage of anterior stromal tissue removed, the spatial profile of the laser ablation, and the residual stromal bed thickness—all of which collectively determine the biomechanical stability of the cornea following LASIK [11,14].

In recent years, investigations have focused on delineating the relative contributions of specific surgical and anatomical variables—such as flap thickness (FT), ablation depth (AD), preoperative central corneal thickness (CCT), percentage of tissue altered (PTA), and residual stromal bed thickness (RST)—to the postoperative biomechanical status of the cornea following LASIK [15,16].

However, numerous studies assessing changes in ORA-derived biomechanical parameters after laser refractive surgery have yielded inconsistent findings [2,8,17].

While several authors have reported a more pronounced influence of FT compared to AD on postoperative corneal biomechanics [18,19], other research challenges this notion. For instance, Uzbek et al. [6] analyzed the changes in CH and CRF at various stages of the FS-LASIK procedure and concluded that flap creation alone does not significantly alter CH or CRF. Their findings suggest that excimer laser ablation, rather than flap formation, is the primary driver of biomechanical changes. Supporting this perspective, additional studies have found a significant correlation between AD and reductions in ORA-measured biomechanical parameters following myopic LASIK [15,20,21,22]. Furthermore, some authors have suggested that the percentage of anterior corneal tissue altered during LASIK (PTA), calculated as (FT + AD)/CCT, is a more robust predictor of postoperative reduction in CH and CRF than any individual component variable [14,18]. In contrast, Kirwan and O’Keefe [21] found that postoperative CCT exerted a greater influence on CH than AD alone. Numerous other studies have emphasized the critical importance of residual stromal bed thickness (RST), identifying it as a principal determinant of biomechanical integrity following LASIK [19,21,23].

The influence of FT on postoperative biomechanical parameters is still discussed. Some authors have observed that increased flap thickness in both MK-LASIK and FS-LASIK procedures is associated with a greater reduction in corneal biomechanical strength as measured by ORA parameters [24]. However, some experimental and clinical studies have also shown that thinner flaps in LASIK do not necessarily correspond to decreased corneal biomechanics [19,21].

The primary objective of this study was to compare refractive and corneal biomechanical parameters measured by ORA during a 6-month follow-up after myopic FS-LASIK with different flap thicknesses (110 µm and 140 µm) and similar ablation ranges. In addition, the study aimed to determine which corneal structural variables and surgical descriptors were significantly associated with biomechanical changes detected by ORA that could significantly predict changes in these biomechanical parameters.

As the anterior 40% of the central corneal stroma exhibits approximately 50% greater strength than the posterior 60% of the stroma [25], and interlamellar cohesive force decreases with stromal depth, two empirically weighted biomechanical indices were used to adjust AD and RST for variation in FT and CCT. The anterior weighted biomechanical index (AWBI) was developed to reflect the cumulative biomechanical impact of both flap creation and laser stromal ablation, normalized by preoperative CCT [22]. In contrast, the posterior weighted biomechanical index (PWBI) was used to account for the biomechanical relevance of the residual stromal thickness also weighted by preoperative CCT [3]. These indices were intended to more accurately capture the biomechanical implications of tissue redistribution and removal, taking into account the differential strength characteristics of the anterior versus posterior cornea. To the best of our knowledge, this study represents the first prospective 6-month comparative analysis investigating the influence of multiple corneal structural parameters on changes in CH and CRF following myopic FS-LASIK with different flap thicknesses (110 µm and 140 µm) and similar ranges of ablation.

## 2. Materials and Methods

This prospective, comparative, single-center study was approved by the Institutional Review Board of the Military Institute of Medicine in Warsaw (Protocol No. 48/WIM/2015) and conducted in accordance with the tenets of the Declaration of Helsinki. All patients signed informed consent forms. Patients of both sexes, aged 18 years or older, with stable refractive status (change not exceeding ± 0.50 D in the past 12 months) were recruited from October 2015 to April 2016 at the Optegra Clinic in Warsaw, Poland.

Inclusion criteria were: preoperative spherical refraction greater than −0.50 diopters (D) but less than −8.00 D, cylindrical refraction up to 2.00 D, preoperative corrected distance visual acuity (CDVA) of 0.5 Snellen or better, central corneal thickness (CCT) measured by pachymetry greater than 500 microns (µm), presumed postoperative residual stromal thickness (RST) greater than 300 µm, and willingness to participate and comply with follow-up. Exclusion criteria included keratoconus or suspected keratoconus, severe dry eye, active or chronic ocular diseases, history of ocular trauma or surgery, autoimmune diseases, pregnancy, and breastfeeding. When both eyes were eligible, data from each eye were regarded independently for the purpose of the analysis.

All patients underwent a comprehensive preoperative ophthalmic assessment, including autokeratorefractometry (Visuref, Carl Zeiss, Oberkochen, Germany), manifest refraction, Snellen uncorrected (UDVA) and corrected (CDVA) distance visual acuities (manual phoropter RX, Reichert, New York, NY, USA; optotype projector FR 1003 LED, Frey SJ, Piaseczno, Poland), intraocular pressure (IOP) by non-contact tonometry (NT-530P, NIDEK, Tokyo, Japan), slit-lamp biomicroscopy and dilated funduscopic examination (90 D Volk lens), cycloplegic refraction, Scheimpflug-based corneal tomography (Pentacam HR; Oculus Optikeräte, Inc., Wetzlar, Germany), Placido-disk topography (Humphrey Atlas, Carl Zeiss Meditec AG, Jena, Germany), wavefront aberrometry (Wasca, Carl Zeiss Meditec, Dublin, CA, USA), ocular biometry (IOL Master 500, Carl Zeiss, Jena, Germany), and dynamic bidirectional applanation tonometry (Ocular Response Analyzer, Reichert, Buffalo, NY, USA). Ocular surface and tear break-up time (BUT) were evaluated after fluorescein instillation. Tear secretion was assessed following topical administration of 0.5% proxymetacaine hydrochloride (Alcaine, Alcon, Belgium). All contact procedures were performed at the end of the examination to avoid affecting other measurements.

ORA measurements included corneal hysteresis (CH), corneal resistance factor (CRF), corneal-compensated IOP (IOPcc), and Goldmann-correlated IOP (IOPg). Three high-quality measurements were obtained with 15 s intervals, and the average was calculated. All preoperative assessments were completed within two weeks prior to surgery.

### 2.1. Surgical Procedure and Postoperative Care

All femtosecond laser-assisted in situ keratomileusis (FS-LASIK) procedures were performed by a single experienced surgeon (M.S.) using the LDV Z4 femtosecond laser system (Ziemer Ophthalmic Systems AG, Switzerland) and MEL 80 excimer laser (Carl Zeiss Meditec AG, Jena, Germany). A standard surgical protocol was followed for all subjects.

Flap thickness with a superior hinge was designed to be either 110 or 140 µm in both eyes. Randomization was done using a coin toss by the same researcher (M.S.), with heads indicating a 110 µm flap thickness and tails indicating a 140 µm flap thickness. Randomization was performed at the patient level, and in all cases, both eyes underwent surgery with the same assigned flap thickness applied to each eye The flap diameter was 9.0 mm with a hinge width of 3.8 mm. A 6.5 mm optical zone with corneal vertex axis-centered spherocylindrical ablation (SCA) was used. All patients were targeted for emmetropia using a surgeon-specific nomogram.

After applying topical 0.5% proxymetacaine (Alcaine, Alcon Laboratories, Puurs-Sint-Amands, Belgium) to the conjunctival sac and inserting an eyelid speculum, the lamellar corneal flap was created using the Femto LDV Z4 laser. The flap was gently lifted, and photoablation was performed with the MEL 80 excimer laser. The flap and stromal bed were then irrigated with balanced salt solution, and the flap was repositioned and smoothed.

Topical 0.5% levofloxacin (Oftaquix; Santen Oy, Tampere, Finland) was instilled, followed by placement of a bandage contact lens. Postoperatively, 0,5% levofloxacin and 0.5% loteprednol eye drops (Lotemax; Dr. Gerhard Mann Chem.-pharm., Berlin, Germany) were administered every 2 h for 1 day, then four times a day for 1 week. Levofloxacin was then discontinued, and loteprednol was tapered over 3 weeks. Preservative-free lubricants were used 4–6 times daily for 3 months.

Ablation depth (AD) and residual stromal thickness (RST) were obtained from the excimer laser system. RST was calculated as preoperative CCT minus the sum of predicted AD and flap thickness (FT). Pre- and postoperative CCT measurements were obtained using the Pentacam HR. The anterior weighted biomechanical index (AWBI) was calculated as the decimal fraction of tissue altered: (FT + AD)/CCT. The AWBI is mathematically equivalent to previously reported indices such as the Percentage of Tissue Altered (PTA) [18] and the Ablation Depth Index (AD Index) [3]. The posterior weighted biomechanical index (PWBI) was calculated as the decimal fraction of tissue remaining intact: RST/CCT.

Follow-up examinations were performed at 1 day, 7 days, 1 month, 3 months, and 6 months postoperatively. Manifest refraction spherical equivalent (MRSE), UDVA, and slit-lamp examination were conducted at all visits. CDVA, BUT, Schirmer II, corneal biomechanics (CH, CRF), IOPg, and IOPcc were assessed at day 7, and at months 1, 3, and 6. Scheimpflug corneal tomography was repeated at 6 months. Efficacy (postoperative UDVA/preoperative CDVA) and safety (postoperative CDVA/preoperative CDVA) indices were calculated for each time point.

Changes in corneal biomechanics were quantified as absolute decreases in CH and CRF (CH drop, CRF drop), percentage changes relative to baseline (CH change, CRF change), and changes in CH and CRF per micrometer of laser ablation (Δ CH/µm, Δ CRF/µm).

Intraoperative and postoperative complications were recorded and compared between the study groups.

### 2.2. Statistical Analyses

Statistical analyses were performed using SAS software (version 9.4). Data normality was assessed with the Shapiro–Wilk test. Normally distributed quantitative variables were presented as mean ± standard deviation (SD), whereas non-normally distributed variables were reported as median (minimum–maximum). Between-group comparisons for continuous variables were performed using the two-tailed unpaired Student’s *t*-test, and within-group changes were analyzed with a paired *t*-test. Categorical variables were compared using the chi-square test. For repeated-measures data with more than two time points, analysis of variance (ANOVA) with Sidak adjustment for multiple comparisons was applied. A multivariate linear regression model with stepwise selection (REG procedure) was used to identify significant predictors of changes in corneal hysteresis (CH) and corneal resistance factor (CRF) from variables including flap thickness (FT), ablation depth (AD), residual stromal bed thickness (RST), central corneal thickness (CCT), anterior weighted biomechanical index (AWBI), and posterior weighted biomechanical index (PWBI). In addition, a generalized estimating equation (GEE) model was applied to account for within-subject clustering due to inclusion of both eyes from each patient, in order to verify the robustness of the regression results. An additional subgroup analysis was also performed to refine predictors of changes in CH and CRF, based on ablation depth (AD), stratifying the sample into two groups: AD < 60 µm and AD ≥ 60 µm, as well as on preoperative central corneal thickness (CCT), dividing patients into two groups: CCT < 540 µm and CCT ≥ 540 µm. Statistical significance was set at *p* < 0.05.

## 3. Results

A total of 186 patients (278 eyes), comprising 118 females and 68 males with a mean age of 28.78 ± 5.3 years and mean manifest refraction spherical equivalent (MRSE) of −4.84 ± 1.4 diopters (D), were initially recruited and treated. Twenty patients were lost to follow-up and excluded. Ultimately, 129 eyes (88 patients; 57 females, 31 males) in the 110 µm flap group (group 110) and 117 eyes (78 patients; 49 females, 29 males) in the 140 µm flap group (group 140) completed the study protocol and were analyzed. Baseline characteristics of both groups are summarized in Table 1. There were no significant differences between groups in terms of age, sex, preoperative refractive errors, mean keratometry, CCT, biomechanical parameters measured by ORA, predicted AD, RST, AWBI, and PWBI. The parameters relevant to surgery are detailed in Table 1.

### 3.1. Refractive Outcomes

**Figure 1:**
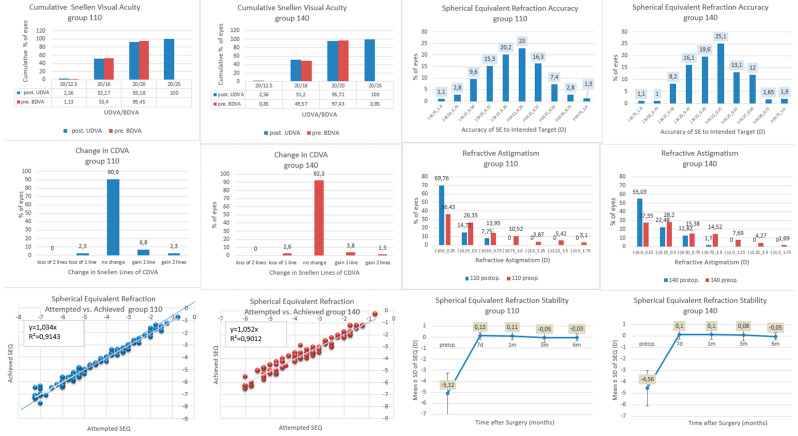
Refractive outcomes of FS-LASIK with two different flap thicknesses (110 µm and 140 µm). Postop = postoperatively; preop = preoperatively; UDVA = uncorrected distance visual acuity; CDVA = corrected distance visual acuity; D = diopter; SEQ = spherical equivalent; d = days; m = months.

No significant differences were observed in uncorrected distance visual acuity (UDVA) or corrected distance visual acuity (CDVA) at any postoperative time point between groups 110 and 140. The mean efficacy index (EI) and safety index (SI) at 6 months postoperatively did not differ significantly between groups. At 6 months, 97.7% of eyes in group 110 and 97.4% of eyes in group 140 achieved CDVA equal to or better than preoperative CDVA. No eyes lost two or more lines of CDVA. Postoperative MRSE was within ± 0.5 D in 93.1% of eyes in group 110 and 94.1% of eyes in group 140, and within ± 1.0 D in 100% of eyes in both groups. The proportion of eyes with postoperative astigmatism ≤ 0.5 D cylinder was 84.48% in group 110 and 85.44% in group 140. Linear regression analyses of attempted versus achieved spherical equivalent yielded slopes of 1.034 (R^2^ = 0.9143) for group 110 and 1.052 (R^2^ = 0.9012) for group 140. Refraction remained stable at 6 months with a mean SE of −0.03 D and −0.05 D, respectively. The refractive outcomes in both study groups are presented in Table 2 and in Figure 1.

### 3.2. Complications

No intraoperative complications occurred. Peripheral microstriae, insignificant for visual acuity or patient discomfort, were present in 6.2% (8 eyes) of group 110 and 4.2% (5 eyes) of group 140 (*p* = 0.162). No cases of diffuse lamellar keratitis or epithelial ingrowth were observed. Corneal fluorescein staining exceeding 15 points was significantly more frequent in group 140 (12.8%, 15 eyes) versus group 110 (3.1%, 4 eyes) at 6 months (*p* < 0.0001). Tear break-up time (TBUT) was also significantly lower in group 140 compared to group 110 (6.43 s vs. 7.72 s, *p* < 0.0001).

### 3.3. Biomechanical Outcomes

Table 3 presents the comparative analysis of corneal biomechanical parameters measured with ORA before and 6 months after FS-LASIK. Both groups exhibited significant decreases in CH and CRF following surgery. On postoperative day 7, mean CH decreased to 9.13 ± 3.26 (*p* = 0.039) and mean CRF to 8.67 ± 2.12 (*p* = 0.029) in group 110; likewise, CH dropped to 8.98 ± 2.23 (*p* = 0.027) and CRF to 8.34 ± 2.64 (*p* = 0.012) in group 140. Subsequent follow-ups at 1, 3, and 6 months showed further, albeit statistically insignificant, decreases in CH and CRF. Over 6 months, mean CH and CRF reductions amounted to 17.9% and 25.9% in group 110 and 26.64% and 32.19% in group 140, respectively.

At 6 months, mean CH was significantly lower in group 140 compared to group 110 (8.30 vs. 8.78, *p* = 0.041), whereas mean CRF did not significantly differ (7.71 vs. 7.83, *p* = 0.09). Absolute and relative reductions in CH and CRF were significantly greater in group 140. Mean reductions per micron of ablation (ΔCH/µm and ΔCRF/µm) were significantly greater in group 140 (0.040 and 0.050) compared to group 110 (0.026 and 0.035) (*p* = 0.001 for ΔCH/µm and *p* = 0.001 for ΔCRF/µm).

At 6 months postoperatively, mean Goldmann-correlated intraocular pressure (IOPg) was 10.37 ± 1.33 mmHg in group 110 (*p* = 0.019) and 11.64 ± 1.93 mmHg in group 140 (*p* = 0.002). Corneal-compensated intraocular pressure (IOPcc) was 13.88 ± 2.20 mmHg and 13.98 ± 1.86 mmHg, respectively. No statistically significant intergroup differences in IOPg or IOPcc were observed.

Multiple regression analysis identified AWBI as the strongest predictor of CH reduction (R^2^ = 0.601 for absolute drop; R^2^ = 0.678 for percentage change), while FT demonstrated moderate explanatory power (R^2^ = 0.311 and 0.376, respectively). Reduction in CRF correlated with FT, RST, AD, and CCT, with FT showing moderate influence (R^2^ = 0.282 for absolute drop; R^2^ = 0.430 for percentage change), whereas AD, RST, and CCT had lower explanatory contributions (R^2^ ranging from 0.051 to 0.298). The results of multiple regression analysis between corneal surgical parameters and changes in biomechanical parameters after FS-LASIK are presented in Table 4.

The GEE analysis revealed that the estimated within-subject correlation (correlation coefficient) was low or modest (ranged from 0.152 to 0.339), suggesting limited inter-eye dependence in the analyzed outcomes. Model fit was evaluated using the Quasi-likelihood under the Independence Model Criterion (QIC) and the corrected version (QICu), both of which indicated stable and adequate model performance. The results from the GEE analysis for all variables are presented in Table 5.

An additional subgroup analysis, based on AD (AD < 60 µm and AD ≥ 60 µm), as well as on preoperative CCT (CCT < 540 µm and CCT ≥ 540 µm), did not reveal any substantial differences in outcomes or identify new predictors of biomechanical changes across these subgroups. The main associations between dependent and independent variables remained consistent with those reported in the overall analysis. The results of these analyses are presented in Table S1 (in Supplementary File).

## 4. Discussion

This prospective study assessed and compared clinical and corneal biomechanical outcomes following myopic femtosecond laser-assisted LASIK (FS-LASIK) using the Ocular Response Analyzer (Reichert, Buffalo, NY, USA) in eyes treated with two different flap thicknesses but similar ablation ranges over a six-month follow-up.

### 4.1. Impact of Flap Thickness on Refractive Outcomes

Our findings indicate that flap thickness did not significantly influence the efficacy, safety, or predictability of FS-LASIK for the correction of myopia. Visual acuity and refractive outcomes were comparable between the 110 µm and 140 µm flap groups throughout the 6-month follow-up period. At six months postoperatively, the mean uncorrected distance visual acuity (UDVA) and corrected distance visual acuity (CDVA) were 0.96 ± 0.04 and 1.02 ± 0.03 in the 110 µm group, and 0.97 ± 0.06 and 1.01 ± 0.03 in the 140 µm group, respectively. Similarly, the mean spherical equivalent (SE) was −0.03 ± 0.46 D and −0.05 ± 0.33 D, respectively. These results align with previous studies. Lim et al. [26] reported no significant differences in UDVA, CDVA or SE outcomes between groups with 80 µm and 120 µm flaps. Likewise, in a retrospective six-month analysis, Eleftheriadis et al. [27] observed slightly faster visual recovery and marginally lower postoperative myopic SE in patients undergoing thin-flap LASIK; however, both the mean UDVA and CDVA at six months were comparable regardless of flap thickness.

Prandi et al. [28] evaluated refractive outcomes across three flap thickness subgroups (<100 µm, 100–130 µm, >130 µm) and reported superior UDVA in eyes with the thinnest flaps, though CDVA remained statistically similar across all groups at six months. In the present study, the efficacy index (EI) did not differ significantly between the thin- and thick-flap groups at any postoperative time point. These findings are in line with previous reports by Han et al. [29] and Brar et al. [30], although the observed EI values in our study were slightly lower than those reported by Lin et al. [31]. Predictability, defined as the percentage of eyes achieving a MRSE within ± 0.5 D of the intended correction, was also comparable between groups (93.0% in the 110 µm group vs. 94.1% in the 140 µm group). Additionally, no significant differences were observed in the rates of under-correction or overcorrection between study groups, further supporting the conclusion that flap thickness, within this tested range, does not adversely affect the refractive precision of FS-LASIK.

The safety index (SI) likewise demonstrated no significant differences between the two cohorts throughout the follow-up period, with values of 1.05 ± 0.39 and 1.03 ± 0.43 six months postoperatively for the 110 µm and 140 µm flap groups, respectively. These values are consistent with those reported by Lin et al. [31], while other studies have noted slightly higher indices ranging from 1.08–1.12 ± 0.16 [30] to 1.19 ± 0.17 [29]. In the present study, no eyes in either group experienced a loss of two or more Snellen lines. A one-line reduction in corrected distance visual acuity (CDVA) was observed in two eyes per group (1.6–1.7%), which is slightly lower than the rates reported elsewhere [32].

The incidence of flap microstriae was slightly higher in the thin-flap group (6.4%) compared to the thick-flap group (4.2%), although this difference was not statistically significant. These findings are in agreement with those reported by Khokhar et al. [33]. Nonetheless, in all cases, microstriae were mild and did not impact visual acuity or require surgical intervention.

### 4.2. Impact of Flap Thickness on Corneal Biomechanics

This study demonstrated that the reduction in corneal biomechanical parameters, as measured by the Ocular Response Analyzer (ORA), was significantly less pronounced in the thin-flap (110 µm) compared to the thick-flap (140 µm) FS-LASIK group. This disparity may be attributed to the greater preservation of anterior stromal integrity in eyes with thinner flaps. Another contributing factor may be the slightly greater residual stromal bed thickness (RST) observed in the thin-flap cohort. Although the predicted RST was slightly higher in the 110 µm flap group, this difference between study groups did not reach statistical significance, suggesting that preservation of anterior stromal tissue may play a more dominant role than absolute RST alone in maintaining postoperative biomechanical strength.

The heterogeneity of corneal biomechanics arises primarily from variations in the organization and density of collagen lamellae, as well as the distribution of keratocytes across the stromal depth. It has been well established that Bowman’s membrane and the anterior 40% of the central corneal stroma represent the biomechanically strongest regions of the cornea, while the posterior 60% exhibits approximately half the tensile strength [25,34,35]. Consequently, surgical techniques that minimize disruption to the anterior stromal layers may confer superior biomechanical preservation. Thicker flaps may lead to greater biomechanical weakening, as they involve deeper dissection into the anterior stroma—compromising the most structurally robust region of the cornea—and simultaneously result in a thinner residual stromal bed, further reducing postoperative corneal stability.

Corneal hysteresis (CH) reflects the cornea’s dynamic viscous response to an applied force, while the corneal resistance factor (CRF) represents its elastic properties and serves as an indicator of overall corneal stiffness and structural strength. Notably, CRF captures the cornea’s static response to deformation, largely independent of the duration of the applied pressure stimulus [20,36].

In the present cohort, there were no statistically significant differences between the 110 µm and 140 µm flap groups in terms of baseline demographic or ocular characteristics, including age, preoperative central corneal thickness (CCT), mean keratometry (K), CH, CRF, spherical equivalent (SE), or planned ablation depth (AD). It has been previously shown that both CH and CRF are influenced by age, CCT, and corneal curvature (K) [37].

Preoperative CH and CRF values in both groups were within normative ranges previously reported in healthy eyes (9.6–12.7 mmHg and 9.9–11.9 mmHg, respectively) [38]. Following FS-LASIK, both CH and CRF decreased significantly in each group, with greater reductions observed in the thick-flap group (−2.89 ± 1.13 vs. −2.04 ± 1.43 mmHg for CH; −3.61 ± 1.32 vs. −2.77 ± 1.46 mmHg for CRF). These results are in line with earlier studies indicating that laser refractive procedures typically result in a 1–3 mmHg decline in both CH and CRF, with the degree of change influenced by factors such as the type of procedure, ablation depth and baseline biomechanical status of the cornea values of these parameters.

Goussous et al. [24] reported similar outcomes in eyes undergoing MK-LASIK with planned flap thicknesses of 90 µm and 130 µm. Their study demonstrated a statistically significant difference in CH reduction: −1.66 ± 1.00 mmHg (15.7%) in the thin-flap group versus 2.62 ± 1.63 mmHg (23.7%) in the thick-flap group (*p* = 0.012). In contrast, reductions in CRF—2.77 ± 1.03 mmHg (26.7%) and 2.92 ± 1.10 mmHg (27.4%)—did not differ significantly between groups (*p* = 0.681), although the overall trend mirrored that of CH.

An experimental study by Medeiros et al. [19] investigated the effect of flap thickness on corneal biomechanics in 12 swine eyes undergoing femtosecond laser-assisted LASIK (FS-LASIK). The eyes were divided into two groups based on flap thickness: a thin-flap group (approximately 100 µm) and a thick-flap group (approximately 300 µm). The results revealed that corneal hysteresis (CH) and corneal resistance factor (CRF) significantly decreased after thick-flap FS-LASIK, with reductions of −2.90 mmHg and −4.10 mmHg, respectively. In contrast, the thin-flap FS-LASIK group exhibited minimal, statistically non-significant changes in CH and CRF (−0.30 mmHg and −0.50 mmHg, respectively), highlighting the biomechanical advantage of preserving the anterior stroma through thinner flap creation.

The reduction in corneal hysteresis (ΔCH) observed in the 110 µm flap FS-LASIK group in the present study is consistent with prior clinical findings. Hamilton et al. [22] documented a mean CH decrease of 1.9 ± 0.9 mmHg following MK-LASIK and FS-LASIK with a 120 µm flap, while Kirwan [21] reported a CH reduction of 1.5 ± 0.5 mmHg in eyes treated with a 100 µm flap. Furthermore, the extent of CRF reduction in the 110 µm flap FS group in the present study (25,9%) was lower than that reported by Hamilton et al. [22] (31.5%), possibly reflecting the 10 µm thinner flap used in our cohort. Notably, in our 140 µm flap group, the CRF reduction (32.2%) closely approximated the value observed in Hamilton’s 120 µm flap cohort (31.5%) [22]. 

In the current study, the reductions in CH and CRF per micron of stromal er ablation (ΔCH/µm and ΔCRF/µm) were significantly greater in the thick-flap group than in the thin-flap group, despite comparable total ablation volumes between the groups. Our findings are consistent with those of Goussous et al. [24], who also reported greater biomechanical weakening per micron of ablation in thicker flap cohorts. In their study, ΔCH/µm was 0.023 in the thin-flap group and 0.049 mmHg/µm in the thick-flap group, while ΔCRF/µm was 0.040 and 0.050 mmHg/µm in the thin- and thick-flap groups, respectively.

In the present study, significant changes in ORA-derived biomechanical parameters were observed as early as 7 days postoperatively, indicating that the majority of biomechanical alteration occurs within the first week following FS-LASIK. Subsequent follow-up evaluations revealed either stabilization or only minor, statistically non-significant additional reductions. These observations are in agreement with prior reports [15], which demonstrated that the most pronounced biomechanical changes occur in the initial postoperative weeks, followed by a plateau phase. Some studies have even reported a partial recovery—up to 10% in CH and CRF values during this stabilization period [39]. Overall, it is widely recognized that reductions in CH and CRF tend to stabilize by approximately 3 months postoperatively and do not return to preoperative baseline levels.

The current study demonstrates that at 6 months following FS-LASIK surgery, both Goldmann-correlated intraocular pressure (IOPg) and corneal-compensated intraocular pressure (IOPcc) were significantly reduced in the 110 µm and 140 µm flap groups. Despite the overall decline in IOPg and IOPcc values, no statistically significant differences in IOPg or IOPcc were observed between the two flap thickness groups, indicating that flap thickness alone may not substantially influence postoperative IOP estimates when ablation volumes are similar. These findings are in agreement with those reported by Medeiros et al. [19].

To better understand the relationship between changes in corneal biomechanical parameters and structural variables, numerous studies have investigated the influence of independent metrics such as central corneal thickness (CCT), ablation depth (AD), and flap thickness (FT). However, given the complex biomechanical behavior of the cornea—particularly in the structurally critical anterior stroma—several composite indices have been introduced to more accurately characterize the biomechanical impact of LASIK. These include the percentage of tissue altered (PTA), as described by Santhiago et al. [40], the ablation depth (AD) index, proposed by Chen [3], and the anterior weighted biomechanical index (AWBI) [22]. In parallel, the residual stromal bed to central corneal thickness ratio (RSB/CCT) has been proposed, used as the residual stromal bed (RSB) index [3], or posterior weighted biomechanical index (PWBI) to assess the structural integrity of the remaining posterior cornea.

Among these indices, PTA—which integrates flap thickness, ablation depth, and preoperative central corneal thickness—has gained particular clinical relevance as a reliable predictor of biomechanical integrity following LASIK. By accounting for the proportion of anterior stromal tissue affected by surgery, PTA offers a more comprehensive assessment of structural impact than any single variable alone.

Smadja et al. [41] demonstrated that the deviation between intended and achieved PTA values was less than 1%, highlighting the high precision and clinical utility of this index in surgical planning. Importantly, a PTA value exceeding 40% has been strongly associated with a significantly increased risk of postoperative corneal ectasia, underscoring its importance as a critical parameter in preoperative risk stratification.

### 4.3. Multivariate Analysis and Comparison to Prior Studies

Our multiple regression analysis identified the anterior weighted biomechanical index (AWBI) and flap thickness (FT) as the most significant predictors of both absolute and relative reductions in corneal hysteresis (CH). AWBI accounted for the highest proportion of variance (R^2^ = 0.601–0.678), underscoring its dominant role in predicting CH reduction. This finding reinforces the importance of stromal depth-weighted indices in predicting postoperative biomechanical behavior. Flap thickness also emerged as a meaningful, though more moderate, predictor of CH reduction, with R^2^ values ranging from 0.311 to 0.376. In contrast, corneal resistance factor (CRF) was influenced by a broader set of structural variables. Significant associations were found between CRF reduction and FT, residual stromal thickness (RST), ablation depth (AD), and central corneal thickness (CCT). Among these, FT again exerted the strongest predictive value, with R^2^ = 0.282 for absolute CRF reduction and R^2^ = 0.430 for relative CRF reduction. While statistically significant, the correlations for AD, RST, and CCT were notably weaker, with R^2^ values ranging from 0.051 to 0.298, suggesting that while these variables contribute to biomechanical weakening, their predictive strength is less consistent compared to FT.

These results collectively emphasize that anterior stromal disruption—quantified by AWBI and directly influenced by FT—is the primary determinant of CH loss, whereas CRF appears to reflect a more multifactorial interaction involving both anterior and posterior corneal structural parameters.

These findings are consistent with those of Chen et al. [3], who examined the correlations between postoperative changes in CH and CRF and various corneal and surgical parameters in the cohort of 43 myopic eyes treated with MK-LASIK with 90 µm or 110 µm flaps. In their study, the authors introduced an ablation depth index analogous to the AWBI and a residual stromal bed (RSB) index conceptually similar to the PWBI used in our study. Chen et al. [3] reported positive correlations between changes in CH and both AD (Pearson r = 0.47, *p* = 0.002) and manifest refraction spherical equivalent (MRSE) (r = 0.51, *p* = 0.005). However, CH was not significantly associated with the AD index. In contrast, changes in CRF showed strong positive correlations with AD (r = 0.65, *p* < 0.0001) and MRSE (r = 0.66, *p* < 0.0001), and were also significantly associated with both the AD and RST indices. These results suggest that CRF may be a more sensitive marker of LASIK-induced biomechanical alterations than CH.

Supporting evidence for the relationship between structural corneal alterations and biomechanical weakening was also provided by Santhiago et al. [42], who assessed ORA-derived biomechanical changes following thin-flap (100–110 µm) FS-LASIK in myopic patients. They reported significant positive correlations between changes in both CH and CRF and ablation depth (Spearman r = 0.379 and 0.452, respectively; both *p* < 0.0001), as well as with the percentage of tissue altered (PTA)—a metric conceptually equivalent to the AWBI used in the present study (r = 0.271 for ΔCH and 0.384 for ΔCRF; both *p* < 0.0001). Similar associations between biomechanical changes (ΔCH, ΔCRF) and PTA were reported by Vanathi et al. [43], with correlation coefficients of –0.39 (*p* = 0.001) for CH and –0.58 (*p* = 0.001) for CRF. Other authors have also found that greater ablation depth and higher percentages of anterior stromal alteration are associated with more pronounced reductions in CH and/or CRF following LASIK [20,21]. In the current study, while AD, RST, and CCT demonstrated weak correlations with reductions in CRF, no statistically significant associations were found with CH. These results are consistent with those reported by Chen et al. [3], who observed moderate correlations between postoperative CRF reductions and both the ablation depth (AD) index (r = 0.47, *p* = 0.001) and the residual stromal bed (RSB) index (r = –0.44, *p* = 0.003), while CH showed no significant relationship with either index.

Vanathi et al. [43] reported that the corneal resistance factor (CRF) exhibits greater sensitivity to biomechanical changes following LASIK compared to corneal hysteresis (CH). As CRF predominantly reflects corneal elasticity and structural stiffness, it may serve as a more robust indicator of LASIK-induced biomechanical alterations. This suggestion is supported by numerous studies showing that CRF tends to decrease more markedly than CH after LASIK. Conversely, Kirwan and O’Keefe [21] observed a strong correlation between postoperative central corneal thickness (CCT) and CH, indicating that CH may be more strongly influenced by postoperative CCT than by the depth of stromal ablation (AD). These findings reinforce the differential sensitivity of CRF and CH to various corneal and surgical factors and highlight their complementary value in assessing corneal biomechanics after refractive surgery.

### 4.4. Wound Healing Considerations

When interpreting the differences in corneal hysteresis (CH) and corneal resistance factor (CRF) parameters between the thin- and the thick-flap FS-LASIK groups, potential differences in wound healing should also be considered. Studies by Wilson et al. [44] demonstrated that greater injury repair activity is more pronounced under thinner corneal flaps, likely due to the increased release of growth factors from keratocytes and myofibroblasts located closer to the epithelium surface. This enhanced cellular activity and inflammatory response in the early postoperative period under thinner flaps may contribute to stronger flap adhesion and greater biomechanical reinforcement in the corneal interface compared to the eyes with thicker flaps. A report by Sonigo et al. [45] similarly suggests that early postoperative inflammation and keratocyte activation contribute to stronger flap adhesion and biomechanical integrity in corneas with thinner flaps. Thus, wound healing dynamics and keratocyte-mediated repair may partly explain the biomechanical differences observed between thin- and thick-flap FS-LASIK groups.

Previous studies have not conclusively determined whether corneal hysteresis (CH) or corneal resistance factor (CRF) serves as the more valuable metric for assessing biomechanical changes following MK-LASIK or FS-LASIK. The literature presents divergent views on this issue. Some authors argue that CH, as a measure of the cornea’s viscoelastic damping capacity, provides greater sensitivity to subtle biomechanical alterations. Conversely, other researchers advocate for CRF as the more comprehensive and reliable indicator, as it reflects a combination of elastic and viscous properties of the cornea. Further research—particularly large-scale, longitudinal studies incorporating advanced dynamic imaging and modeling techniques—is needed to clarify the relative predictive value of CH and CRF, and to determine their optimal application and new metrics in risk stratification and surgical planning in corneal refractive surgery.

### 4.5. Study Limitations

This study had several limitations. First, the inclusion of both eyes from the same participants in the statistical analysis may introduce inter-eye correlation, potentially violating the assumption of statistical independence. This could affect the robustness of certain comparisons and may lead to an underestimation of variability within the dataset. To address this limitation, the authors conducted an additional GEE analysis, which provided further statistical insight and enhanced the credibility of the results. Second, the accuracy of the femtosecond laser flap cutting depth and residual stromal thickness (RST) was not verified intra- or postoperatively using imaging modalities such as anterior segment optical coherence tomography (AS-OCT) or ultrasonic pachymetry. Although this is a limitation, prior studies have consistently demonstrated that femtosecond laser-assisted flap creation is highly accurate and reproducible, with deviations from the intended flap thickness typically within ±5 µm [23,46,47]. Third, the biomechanical assessment relied solely on the Ocular Response Analyzer (ORA), whereas combining ORA with complementary technologies such as the Corvis ST, which captures dynamic corneal response and deformation patterns, would allow for a more comprehensive evaluation of corneal biomechanical changes following FS-LASIK. Despite these limitations, the study’s large sample size, prospective design, and use of matched surgical groups represent important methodological strengths. These factors contribute meaningful insights into the biomechanical behavior of the cornea following FS-LASIK with varying flap thicknesses.

## 5. Conclusions

In summary, femtosecond laser-assisted LASIK (FS-LASIK) performed with different flap thicknesses (110 µm and 140 µm) resulted in equally effective and safe refractive outcomes for the correction of myopia and myopic astigmatism. This prospective study provides further evidence that thicker FS-LASIK flaps exert a more substantial impact on corneal biomechanical integrity compared to thinner flaps. Among the evaluated structural parameters, flap thickness was found to influence postoperative biomechanical changes more significantly than ablation depth alone. The anterior weighted biomechanical index (AWBI) emerged as the most robust predictor of CH reduction, highlighting the value of depth-weighted composite indices in capturing the biomechanical consequences of anterior stromal alteration. Future research is warranted to establish optimized FS-LASIK planning parameters that ensure both refractive efficacy and long-term corneal stability.

## Figures and Tables

**Table 1:** Baseline characteristics.

Characteristic	Group 110	Group 140	*p*- Value
Eyes (patients)	129 (88)	117 (78)	0.092
Age (y), mean ± SD	28.35 ± 6.69	27.89 ± 6.97	0.540 ^a^
Sex; no. male; (%)/no. female; (%)	31; (35.22)/57; (64.77)	29; (31.18)/49; (62.82)	0.123 ^b^
SE (D), mean ± SD	−5.12 ± 1.86	−4.56 ± 1.56	0.064 ^a^
UDVA, mean ± SD	0.048 ± 0.27	0.053 ± 0.25	0.087 ^a^
CDVA, mean ± SD	0.97 ± 0.06	0.98 ± 0.08	0.763 ^a^
Avg K (D), mean ± SD	43.12 ± 1.34	42.56 ± 1.65	0.056 ^a^
Pachymetry (µm), mean ± SD	555.81 ± 30.50	558.13 ± 30.60	0.467 ^a^
CH (mmHg), mean ± SD	10.72 ± 1.63	11.13 ± 1.47	0.087 ^a^
CRF (mmHg), mean ± SD	10.67 ± 1.69	11.32 ± 1.74	0.057 ^a^
IOPg (mmHg), mean ± SD	15.61 ± 2.34	16.54 ± 2.69	0.765 ^a^
IOPcc (mmHg), mean ± SD	15.92 ± 1.57	15.97 ± 2.13	0.956 ^a^
AD (µm), mean ± SD	78.20 ± 24.53	71.89 ± 27.29	0.059 ^a^
AD + FT (µm), mean ± SD	188.2 ± 24.53	211.89 ± 27.20	0.050 ^a^
RST (µm), mean ± SD	338.06 ± 34.15	351.22 ± 34.10	0.053 ^a^
AWBI, mean ± SD	0.38 ± 0.04	0.36 ± 0.05	0.389 ^a^
PWBI, mean ± SD	0.62 ± 0.06	0.64 ± 0.04	0.456 ^a^

^a^ Independent Student’s *t*-test; ^b^ Chi-square (χ^2^) test; y = year; SD = standard deviation; no. = number; D = diopter; SE = spherical equivalent; UDVA = uncorrected distance visual acuity; CDVA = corrected distance visual acuity; Avg K = average keratometry; µm = micrometer; mmHg = millimeter of mercury; CH = corneal hysteresis; CRF = corneal resistance factor; IOPg = Goldmann-correlated intraocular pressure; IOPcc = corneal-compensated intraocular pressure; AD = ablation depth; FT = flap thickness; RST = residual stromal thickness; AWBI = anterior weighted biomechanical index; PWBI = posterior weighted biomechanical index.

**Table 2:** Refractive outcomes.

Characteristic	Group 110	Group 140	*p*-Value
SE (D), mean ± SD	−5.12 ± 1.86	−4.56 ± 1.56	0.064 ^a^
SE 7-d (D), mean ± SD	0.13 ± 0.33	0.10 ± 0.36	0.876 ^a^
SE 6-m (D), mean ± SD	−0.03 ± 0.46	−0.05 ± 0.33	0.754 ^a^
UDVA, mean ± SD	0.048 ± 0.27	0.053 ± 0.25	0.087 ^a^
UDVA 7-d, mean ± SD	0.90 ± 0.10	0.87 ± 0.13	0.353 ^a^
UDVA 1-m, mean ± SD	0.92 ± 0.08	0.95 ± 0.07	0.273 ^a^
UDVA 3-m, mean ± SD	0.95 ± 0.08	0.98 ± 0.07	0.454 ^a^
UDVA 6-m, mean ± SD	0.96 ± 0.04	0.97 ± 0.06	0.767 ^a^
CDVA, mean ± SD	0.97 ± 0.06	0.98 ± 0.08	0.763 ^a^
CDVA 1-d, mean ± SD	0.92 ± 0.06	0.93 ± 0.12	0.561 ^a^
CDVA 1-m, mean ± SD	1.02 ± 0.09	1.02 ± 0.07	0.452 ^a^
CDVA 3-m, mean ± SD	1.02 ± 0.05	1.01 ± 0.04	0.795 ^a^
CDVA 6-m, mean ± SD	1.02 ± 0.03	1.01 ± 0.03	0.799 ^a^
EI 7-d, mean ± SD	0.92 ± 0.24	0.88 ± 0.22	0.534 ^a^
EI 1-m, mean ± SD	0.95 ± 0.18	0.97 ± 0.15	0.789 ^a^
EI 3-m, mean ± SD	0.98 ± 0.15	1.00 ± 0.17	0.734 ^a^
EI 6-m, mean ± SD	0.99 ± 0.14	0.99 ± 0.16	0.816 ^a^
SI 7-d, mean ± SD	0.95 ± 0.34	0.95 ± 0.29	0.987 ^a^
SI 1-m, mean ± SD	1.05 ± 0.32	1.04 ± 0.32	0.945 ^a^
SI 3-m, mean ± SD	1.05 ± 0.35	1.03 ± 0.36	0.912 ^a^
SI 6-m, mean ± SD	1.05 ± 0.39	1.03 ± 0.43	0.878 ^a^

^a^ Independent Student’s *t*-test; SE = spherical equivalent; D = diopter; SD = standard deviation; d = days; m = months; UDVA = uncorrected distance visual acuity; CDVA = corrected distance visual acuity; EI = efficacy index; SI = safety index.

**Table 3:** Comparison of biomechanical parameters of the eyes that underwent FS-LASIK.

Characteristic	Group 110	Group 140	*p*-Value
CH 6-m (mmHg), mean ± SD	8.78 ± 2.04	8.30 ± 1.69	0.041 ^a^
CH drop 6-m (mmHg), mean ± SD	2.04 ± 1.43	2.89 ± 1.13	0.012 ^a^
CH change 6-m (%), mean ± SD	17.95 ± 16.67	26.64 ± 10.39	0.001 ^a^
Δ CH/µm 6-m(mmHg), mean	0.026	0.040	0.001 ^a^
CRF 6-m (mmHg), mean ± SD	7.93 ± 1.88	7.71 ± 1.64	0.090 ^a^
CRF drop 6-m (mmHg), mean ± SD	2.77 ± 1.46	3.61 ± 1.32	0.001 ^a^
CRF change 6-m (%), mean ± SD	25.97 ± 13.01	32.19 ± 10.09	0.001 ^a^
Δ CRF/µm 6-m (mmHg), mean	0.035	0.050	0.001 ^a^
IOPg 6-m (mmHg), mean ± SD	10.37 ± 1.33	11.64 ± 1.93	0.674 ^a^
IOPcc 6-m (mmHg), mean ± SD	13.88 ± 2.20	13.98 ± 1.86	0.956 ^a^
Avg K 6-m (D), mean ± SD	38.91 ± 1.12	38.69 ± 1.23	0.987 ^a^
Pachymetry 6-m (µm), mean ± SD	473.43 ± 27.70	480.78 ± 29.60	0.675 ^a^

^a^ Independent Student’s *t*-test; CH = corneal hysteresis; m = months; mmHg = millimeter of mercury; SD = standard deviation; CH drop = absolute drop of corneal hysteresis; CH change = relative drop of corneal hysteresis; Δ CH = change in corneal hysteresis; µm = micrometer; Δ CH/µm = change in corneal hysteresis per each micrometer of ablation; CRF = corneal resistance factor; CRF drop = absolute drop of corneal resistance factor; CRF change = relative drop of corneal resistance factor; Δ CRF = change in corneal resistance factor; Δ CRF/µm = change in corneal resistance factor per each micrometer of ablation; IOPg = Goldmann-correlated intraocular pressure; IOPcc = corneal-compensated intraocular pressure; Avg K = average keratometry; D = diopter.

**Table 4:** Multiple regression analysis between corneal surgical parameters and absolute and relative changes in biomechanical parameters after FS-LASIK.

Model Fit	1.A F = 19.61, *p* < 0.0001					1.B F = 35.68, *p* < 0.0001				
Independent Variable	Parameter Estimate	T-value	*p*-value	(95% CI)	Overall model R^2^	Parameter Estimate	T-value	*p*-value	(95% CI)	Overall model R^2^
	Dependent Variable—CH drop					Dependent Variable—CH change				
AD	0.01079	2.65	0.0085	(0.003–0.019)	0.68					0.74
AWBI	5.44881	2.19	0.0296	(0.572–10.325)		87.28258	6.11	<0.0001	(59.284–115.282)	
FT	0.02804	5.29	<0.001	(0.018–0.038)		0.27607	6.55	<0.0001	(0.193–0.359)	
Model fit	1.C F = 21.19, *p* < 0.0001					1.D F = 28.15, *p* < 0.0001				
Independent Variable	Parameter Estimate	T-value	*p*-value	(95% CI)	Overall model R^2^	Parameter Estimate	T-value	*p*-value	(95% CI)	Overall model R^2^
	Dependent Variable—CRF drop					Dependent Variable—CRF change				
AD	0.01127	2.62	0.0094	(0.003–0.020)	0.63	0.12237	4.19	<0.0001	(0.065–0.179)	0.69
FT	0.02473	4.21	<0.0001	(0.013–0.036)		0.20702	4.58	<0.0001	(0.118–0.296)	
RST	−0.01198	−3.15	0.0018	(−0.019–−0.005)		−0.07293	−3.67	0.0003	(−0.112–−0.034)	
CCT	0.01009	2.20	0.0290	(0.001–0.019)						

F = the value of the model statistic in multiple regression analysis; *p* = probability value; T-value = t-statistic for each individual regression coefficient; R^2^ = coefficient of determination; CH = corneal hysteresis; CH drop = absolute drop of corneal hysteresis; CH change = relative drop of corneal hysteresis; AD = ablation depth; AWBI = anterior weighted biomechanical index; FT = flap thickness; CRF = corneal resistance factor; CRF drop = absolute drop of corneal resistance factor; CRF change = relative drop of corneal resistance factor; RST = residual stromal thickness; CCT = central corneal thickness.

**Table 5:** The generalized estimating equation (GEE) analysis between corneal surgical parameters and absolute and relative changes in biomechanical parameters after FS-LASIK.

2.A	CH Drop—GEE Model Results with Patient-Level Clustering 2 Eyes per Patient					
Independent Variable	Parameter Estimate	Z	*p*-Value	95% CI	Criterion	Value
FT	0.0278	5.16	<0.0001	0.017–0.038	QIC	249.919
AD	0.0097	2.80	0.0052	0.003–0.016	QICu	250.00
AWBI	6.1691	2.77	0.0056	1.801–10.537	Estimated within-patient correlation	0.152
2.B	CH Change—GEE Model Results with Patient-Level Clustering 2 Eyes per Patient					
Independent Variable	Parameter Estimate	Z	*p*-Value	95% CI	Criterion	Value
FT	0.2753	6.36	<0.0001	0.191–0.360	QIC	250.196
AWBI	88.7040	5.26	<0.0001	55.672–121.737	QICu	249.00
					Estimated within-patient correlation	0.152
2.C	CRF Drop—GEE Model Results with Patient-Level Clustering 2 Eyes per Patient					
Parameter	Parameter Estimate	Z	*p*-Value	95% CI	Criterion	Value
FT	0.0297	5.07	0.0001	0.018–0.041	QIC	254.09
AD	0.0164	4.29	<0.0001	0.0089–0.024	QICu	251.00
RST	−0.0056	−2.08	0.0372	−0.011–−0.0003	Estimated within-patient correlation	0.25
2.D	CRF Change—GEE Model Results with Patient-Level Clustering 2 Eyes per Patient					
Independent Variable	Parameter Estimate	Z	*p*-Value	95% CI	Criterion	Value
FT	0.2264	4.47	<0.0001	0.127–0.326	QIC	252.51
AD	0.0724	2.24	0.0253	0.009–0.136	QICu	251.00
RST	−0.0520	−2.20	0.0279	−0.098–−0.006	Estimated within-patient correlation	0.339
AWBI	57.12	2.45	0.0142	11.465–102.775		

CH = corneal hysteresis; CH drop = absolute drop of corneal hysteresis; CH change = relative drop of corneal hysteresis; FT = flap thickness; AWBI = anterior weighted biomechanical index; AD = ablation depth; RST = residual stromal thickness; CRF = corneal resistance factor; CRF drop = absolute drop of corneal resistance factor; CRF change = relative drop of corneal resistance factor; QIC = Quasi-likelihood under the Independence Model Criterion; QICu = corrected Quasi-likelihood under the Independence Model Criterion.

## Data Availability

Datasets supporting this study are available from J.W. and M.S. upon reasonable request.

## Supplementary Materials

The following supporting information can be downloaded at: https://www.mdpi.com/article/10.3390/jcm15051923/s1, Table S1: Multiple regression analysis between corneal surgical parameters and absolute and relative changes in biomechanical parameters after FS-LASIK, stratifying the sample into the subgroups, based on ablation depth and preoperative central corneal thickness.

## Abbreviations

The following abbreviations are used in this manuscript:

MK-LASIK	Microkeratome-assisted laser in situ keratomileusis
FS-LASIK	Femtosecond-laser-assisted laser in situ keratomileusis
KLEX	Keratorefractive lenticule extraction
ORA	Ocular Response Analyzer
CH	Corneal hysteresis
CRF	Corneal resistance factor
FT	Flap thickness
AD	Ablation depth
CCT	Central corneal thickness
PTA	Percentage of tissue depth altered
RST	Residual stromal bed thickness
AWBI	Anterior weighted biomechanical index
PWBI	Posterior weighted biomechanical index
IOPcc	Corneal-compensated intraocular pressure
IOPg	Goldmann-compensated intraocular pressure
UDVA	Uncorrected distance visual acuity
CDVA	Corrected distance visual acuity
EI	Efficacy index
SI	Safety index
