# Supplementary material for: Impact of Flap Thickness on Refractive Outcomes and Corneal Biomechanics Following Myopic Femtosecond Laser-Assisted LASIK
Source: J Clin Med. 2026 Mar 3;15(5):1923. doi: 10.3390/jcm15051923 (PMC12986029; doi:10.3390/jcm15051923)
Supplement: Supplementary file 1 [file jcm-15-01923-s001.zip › jcm-4114875-supplementary.pdf]

Table S1. Multiple regression analysis between corneal surgical parameters and absolute and relative changes in biomechanical parameters after FS-LASIK, stratifying the sample into the subgroups, based on ablation depth and preoperative central corneal thickness.

Model fit	**1.A F = 9.14, p <0.0001**					**1.B F = 12.72, p <0.0001**				
Independent Variable	Parameter Estimate	T-value	p-value	(95% CI)	Overall model R^2	Parameter Estimate	T-value	p-value	(95% CI)	Overall model R^2
	**Dependent Variable - CH drop**					**Dependent Variable - CH change**				
**FT**	0.02525	4.53	<.0001	(0.0143 - 0.0362)	0.21	0.26134	5.96	<.0001	(0.1754 - 0.3473)	0.24
**AWBI**	9.87090	2.28	0.0236	(1.3803 - 18.362)		100.4785	2.95	0.0035	(33.733 - 167.224)	
**PWBI**	2.77934	0.74	0.4589	(–4.563 - 10.122)		27.66610	0.94	0.3485	(–30.061 - 85.393)	
**AD**	0.15578	0.65	0.5177	(–0.3155 - 0.627)		-1.35894	-0.72	0.4729	(–5.0635 - 2.3456)	
**CCT**	0.24750	1.26	0.2086	(–0.1372 - 0.632)		0.35066	0.23	0.8204	(–2.6730 - 3.3743)	
**RST**	-0.00455	-1.58	0.1144	(–0.0102 - 0.001)		-0.04217	-1.87	0.0630	(–0.0864 - 0.0021)	
Model fit	**1.C F = 12.48, p<0.0001**					**1.D F = 13.92, p<0.0001**				
Independent Variable	Parameter Estimate	T-value	p-value	(95% CI)	Overall model R^2	Parameter Estimate	T-value	p-value	(95% CI)	Overall model R^2
	**Dependent Variable - CRF drop**					**Dependent Variable - CRF change**				
**FT**	0.02615	4.57	<.0001	(0.0149 - 0.0374)	0.24	0.19022	4.05	<.0001	(0.0982 - 0.2822)	0.26
**AWBI**	3.90093	0.88	0.3813	(–4.815 - 12.617)		86.72712	2.38	0.0182	(15.238 - 158.22)	
**PWBI**	-1.21917	-0.32	0.7515	(–8.758 - 6.321)		29.60635	0.94	0.3489	(–32.224 - 91.437)	
**AD**	0.31647	1.28	0.2010	(–0.167 - 0.800)		0.41703	0.21	0.8370	(–3.551 - 4.385)	
**CCT**	0.46787	2.32	0.0211	(0.073 - 0.863)		2.86949	1.74	0.0839	(–0.371 - 6.110)	
**RST**	-0.00909	-3.08	0.0023	(–0.015 - – 0.003)		-0.09473	-3.92	0.0001	–0.1421 to – 0.0473	

F- value = the value of the model statistic in multiple regression analysis; p = probability value; T-value = t -statistic for each individual regression coefficient; R^2 = coefficient of determination; CH = corneal hysteresis; CH drop = absolute drop of corneal hysteresis, CH change = relative drop of corneal hysteresis ; FT = flap thickness; AWBI = anterior weighted biomechanical index; PWBI = posterior weighted biomechanical index; AD = ablation depth; CCT = central corneal thickness; RST = residual stromal thickness; CRF = corneal resistance factor; CRF drop = absolute drop of corneal resistance factor; CRF change = relative drop of corneal resistance factor
